# Preclinical activity of allogeneic SLAMF7-specific CAR T-cells (UCARTCS1) in multiple myeloma

**DOI:** 10.1136/jitc-2023-008769

**Published:** 2024-07-25

**Authors:** Charlotte L B M Korst, Chloe O’Neill, Wassilis S C Bruins, Meliha Cosovic, Inoka Twickler, Christie P M Verkleij, Diane Le Clerre, Maria Themeli, Isabelle Chion-Sotinel, Sonja Zweegman, Roman Galetto, Tuna Mutis, Niels W C J van de Donk

**Affiliations:** 1 Department of Hematology, Amsterdam UMC, Vrije Universiteit, Amsterdam, The Netherlands; 2 Cancer Biology and Immunology, Cancer Center, Amsterdam, The Netherlands; 3 Cellectis SA, Paris, France

**Keywords:** Multiple Myeloma, Immunotherapy, Chimeric antigen receptor - CAR, T cell

## Abstract

**Background:**

Autologous BCMA-specific CAR T-cell therapies have substantial activity in multiple myeloma (MM). However, due to logistical limitations and BCMA^low^ relapses, there is a need for alternatives. UCARTCS1 cells are ‘off-the-shelf’ allogeneic CAR T-cells derived from healthy donors targeting SLAMF7 (CS1), which is highly expressed in MM cells. In this study, we evaluated the preclinical activity of UCARTCS1 in MM cell lines, in bone marrow (BM) samples obtained from MM patients and in an MM mouse model.

**Methods:**

Luciferase-transduced MM cell lines were incubated with UCARTCS1 cells or control (non-transduced, SLAMF7/TCRαβ double knock-out) T-cells at different effector to target ratios for 24 hours. MM cell lysis was assessed by bioluminescence. Anti-MM activity of UCARTCS1 was also evaluated in 29 BM samples obtained from newly diagnosed patients (n=10), daratumumab-naïve relapsed/refractory patients (n=10) and daratumumab-refractory patients (n=9) in 24-hour flow cytometry-based cytotoxicity assays. Finally, UCARTCS1 activity was assessed in mouse xenograft models.

**Results:**

UCARTCS1 cells induced potent CAR-mediated, and dose-dependent lysis of both MM cell lines and primary MM cells. There was no difference in ex vivo activity of UCARTCS1 between heavily pretreated and newly diagnosed patients. In addition, efficacy of UCARTCS1 was not affected by SLAMF7 expression level on MM cells, proportion of tumor cells, or frequency of regulatory T-cells in BM samples obtained from MM patients. UCARTCS1 treatment eliminated SLAMF7^+^ non-malignant immune cells in a dose-dependent manner, however lysis of normal cells was less pronounced compared to that of MM cells. Additionally, durable anti-MM responses were observed with UCARTCS1 in an MM xenograft model.

**Conclusions:**

These results demonstrate that UCARTCS1 has potent anti-MM activity against MM cell lines and primary MM cells, as well as in an MM xenograft model and support the evaluation of UCARTCS1 in patients with advanced MM.

**WHAT IS ALREADY KNOWN ON THIS TOPIC**

- Autologous BCMA-specific CAR T-cell therapies have substantial activity in multiple myeloma (MM). However, due to logistical limitations and BCMA^low^ relapses, there is a need for alternatives.
- Several autologous SLAMF7 CAR T-cell products demonstrated anti-MM activity in preclinical models. However, the potential of using allogeneic SLAMF7-specific CAR T cells for MM treatment has not been explored.

**WHAT THIS STUDY ADDS**

- UCARTCS1 is an off-the-shelf SLAMF7-targeting allogeneic CAR T-cell product derived from healthy donors, which can be directly administered to MM patients without the need to wait for a time-consuming manufacturing process. We demonstrate that UCARTCS1 induces strong CAR-mediated and dose-dependent lysis of different MM cell lines and primary MM cells. Activity of UCARTCS1 was independent of SLAMF7 expression level on MM cells, proportion of tumor cells, or frequency of regulatory T-cells in bone marrow samples obtained from MM patients.
- Furthermore, UCARTCS1 cells had comparable antitumor activity in samples from newly diagnosed and heavily pretreated patients, indicating that mechanisms of resistance to prior therapies do not result in reduced susceptibility to UCARTCS1-mediated lysis. On-target/off-tumor toxicity of UCARTCS1 included the elimination of a subset of B cells, NK cells, and T-cells with highest levels of SLAMF7 while sparing cells with low or no SLAMF7 expression.

**HOW THIS STUDY MIGHT AFFECT RESEARCH, PRACTICE OR POLICY**

- This study supports the further evaluation of UCARTCS1 as a novel treatment strategy for patients with heavily pretreated MM. Assessment of UCARTCS1 in humans will provide a better understanding if, and to what extent, UCARTCS1 could induce immune deficiency and increased risk of infections.

## Introduction

 Multiple myeloma (MM) patients who develop disease refractory to immunomodulatory drugs (IMiDs), proteasome inhibitors, and anti-CD38 antibodies have a very poor overall survival of less than 1 year.^1 2^ Adoptive cell therapy with chimeric antigen receptor (CAR) T-cells has emerged as a potent treatment strategy with recent approval of two CAR T-cell therapies (ide-cel and cilta-cel) for the treatment of patients with heavily pretreated MM.^3^,^5^ These BCMA-targeting CAR T-cell products use genetically modified autologous T-cells from each individual patient.^6^ However, difficulties in (timely) generation of CAR T-cells and the limited availability of production slots, emphasize the need for alternative, “off the shelf” available sources of CAR T-cells.

Additionally, although BCMA-targeted T-cell immunotherapies (CAR T-cell therapy and bispecific antibodies) have substantially improved survival of triple-class refractory MM patients, most patients eventually develop disease progression.^3^,^5^ Frequent occurrence of BCMA^low/negative^ disease and presence of BCMA mutations at the time of progression following BCMA-directed therapy underscores the need to investigate alternative target antigens for T-cell immunotherapies.^7^,^12^

SLAMF7 (also known as CD319 or CS1) is a member of the signaling lymphocyte activation molecule family of receptors with both activating and inhibitory effects on immune cells.^13^ This receptor is highly expressed in normal and malignant plasma cells and at low levels in subsets of normal immune cells including T-cells, NK cells and B cells which makes SLAMF7 an attractive target for immunotherapy.^14^,^16^ The role of SLAMF7 in the pathogenesis of MM has not been fully elucidated, but data suggest that SLAMF7 mediates MM cell adhesion to bone marrow stromal cells and plays an important role in MM cell survival.^15^ The potential of targeting SLAMF7 has already been shown by the clinical activity of elotuzumab, a SLAMF7-specific monoclonal antibody, in combination with IMiDs and dexamethasone in patients with relapsed/refractory MM (RRMM).^17^,^19^ In addition, autologous SLAMF7-specific CAR T-cells demonstrated substantial anti-MM activity in preclinical models.^1620^,^22^ However, the use of allogeneic SLAMF7-specific CAR T cells for MM treatment has not been explored in a preclinical setting using patient samples.

UCARTCS1 cells are “off the shelf” allogeneic CAR T-cells derived from healthy donors, engineered to express a CAR targeting SLAMF7. Moreover, the genes encoding for the T-cell receptor (TCR) α chain of the TCRαβ receptor and for SLAMF7 are disrupted in UCARTCS1 cells using transcription activator-like effector nuclease (TALEN) gene-editing technology.^23^ These modifications aim to minimize the potential risk for allo-TCR-mediated graft-versus-host disease (GvHD) and to prevent SLAMF7-mediated fratricide.

In this study, we evaluated the cytotoxic activity of UCARTCS1 cells against MM cell lines, bone marrow (BM) samples obtained from both newly diagnosed MM (NDMM) and heavily pretreated MM patients, and in an MM xenograft model. We also investigated the impact of previous therapy and tumor characteristics, such as cell surface expression of SLAMF7, on the ex vivo efficacy of UCARTCS1.

## Materials and methods

### UCARTCS1 cells

Research-grade UCARTCS1 cells and control cells were generated by Cellectis SA (Paris, France). UCARTCS1 cells are gene-edited T-cells generated from normal healthy donor peripheral blood T-cells. Upon initial stimulation with anti-CD3/CD28 reagents, activated T-cells were electroporated with mRNAs encoding TALEN to inactivate the *TCRα constant chain* (*TRAC*) gene in order to disrupt surface expression of TCRαβ and to minimize the risk of GvHD. The SLAMF7 gene is also inactivated using TALEN technology in order to facilitate robust expansion and yield by preventing fratricide of SLAMF7-specific CAR^+^ T-cells. Vectorization of the coding sequence of the second-generation CAR targeting SLAMF7 (containing a 4-1BB costimulatory domain) was performed using a self-inactivating lentiviral vector, where CAR expression was under transcriptional control of the human elongation factor 1 alpha gene promoter (hEF1α). Cell expansion was performed in OpTmizer medium (Gibco) supplemented with 5% human serum AB (Seralab), Glutamax (Thermo Fisher) and Penicillin-Streptomycin (Fisher Scientific), plus 350 IU/mL IL-2 (Miltenyi Biotech) and enriched with 5% CTS Immune cell serum replacement (CTS Immune Cell SR, Gibco). At the end of the amplification step, the remaining TCRαβ^+^ cells were depleted using a CliniMACS device (Miltenyi Biotech) and the recovered TCRαβ^−^ cellular fraction was cryopreserved. Schematic overview of CAR T-cell production and associated information is presented in online supplemental figure 1.

Batches generated from T-cells obtained from two different healthy donors were used in this study. The percentage of T-cells expressing the SLAMF7 CAR was 50% for donor 1 and 30% for donor 2, and all T-cells were TCRαβ negative (online supplemental figure 2A,B). In cytotoxicity assays number of effector cells used was adjusted for percentage of CAR^+^ cells. Non-transduced, SLAMF7/TCRαβ double knock-out T-cells from the same donor were used as negative control.

### MM cell lines

The luciferase (LUC)-transduced MM cell lines UM9, U266, L363 and MM.1S were cultured in RPMI-1640 (Invitrogen, Carlsbad, California, USA), supplemented with 10% HyClone FetalClone I serum (GE Healthcare Life Sciences, Marlborough, Massachusetts, USA) and antibiotics (100 units/mL penicillin, 100 µg/mL streptomycin). UM9 was obtained after prolonged in vitro culture of the BM aspirate of an MM patient. U226 and MM.1S were purchased from the American Tissue Culture Collection (Manassas, Virginia, USA), and L363 was obtained from the German Collection of Microorganisms and Cell Cultures. Monthly mycoplasma testing was performed using real-time PCR (Microbiome). Cell lines were authenticated by short-tandem repeat profiling carried out maximal 6 months before the most recent experiment. Cell lines were cultured for a time period no longer than 4 months.

### Bioluminescence imaging-based cytotoxicity assays

LUC-transduced MM cell lines with different surface expression levels of SLAMF7 were incubated with UCARTCS1 cells or control (non-transduced, SLAMF7/TCRαβ double knock-out) T-cells at different effector to target (E:T) ratios for 24 hours. MM cell lysis was determined by bioluminescence imaging (BLI), 30 min after addition of the substrate luciferin (150 µg/mL; Promega, Madison, Wisconsin, USA). MM cell lysis was calculated using the following formula: % lysis=1−(mean BLI signal in the treated wells/BLI signal in untreated wells)×100%. BLI signal was corrected for BLI signal in wells containing only phosphate buffered saline (PBS).

### Patient-derived primary MM cells

Anti-MM activity of UCARTCS1 was evaluated in 29 BM samples obtained from NDMM patients (n=10; NDMM), daratumumab-naïve RRMM patients (n=10; RRMM) and daratumumab-refractory MM patients (n=9; DRMM). BM mononuclear cells (BM-MNCs) from BM aspirates were isolated by Ficoll-Hypaque density centrifugation and cryopreserved in liquid nitrogen until use.

### Cytogenetic analysis of primary MM cells

Cytogenetic abnormalities were assessed in CD138^+^ purified MM cells by fluorescence in situ hybridization (FISH) and by single nucleotide polymorphism array. High-risk disease was defined by the presence of del(17p), t(4; 14) or t(14;16).^24^ Gain/amp 1q was separately evaluated because the gene encoding SLAMF7 is located on the long arm of chromosome 1 at 1q23-24.

### Flow cytometric analysis of BM samples from MM patients

Cryopreserved BM samples were thawed and incubated overnight at 37^◦^C before baseline flow cytometric analysis, which included several tumor characteristics and immune cell composition. Specifically, plasma cells and other immune cell subsets were identified and analyzed for cell surface marker expression levels by staining 0.5–1.0×10^6^ cells with CD45-KO, CD56 PC7, CD138 PE (Beckman Coulter), CD38-multi-epitope-FITC (Cytognos), CD3 BUV395, CD4 BUV737, CD8 BUV496, CD14 BB700, CD19 PE-CF594, CD127 PE-C7, CD319 (SLAMF7) AF647 (all BD Biosciences) and CD25 PE (DAKO). Regulatory T-cells (Tregs, CD3+CD4+CD25+CD127^−/low^) were additionally identified in a subset of BM samples, whenever sufficient material was available.

Flow cytometry was performed using a five-laser LSRFORTESSA (BD Biosciences). Fluorescent-labeled beads (CS&T beads, BD Biosciences) were used daily to monitor the performance of the flow cytometer and verify optical path and stream flow. This procedure enables controlled standardized results and allows the determination of long-term drifts and incidental changes within the flow cytometer. No changes were observed which could affect the results. Compensation beads were used to determine spectral overlap, compensation was automatically calculated using FACSDiva software (BD Biosciences). Flow cytometry data were analyzed using FCS Express 7 Flow software (De Novo Software, Pasadena, California, USA). SLAMF7 levels were expressed as median fluorescence intensity (MFI) values.

### Flow cytometry-based ex vivo cytotoxicity assay

BM-MNCs derived from 29 MM patients, containing tumor cells, as well as stromal cells, autologous effector cells and immune suppressive cells, were used in flow cytometry-based lysis assays. BM-MNCs were incubated in RPMI-1640 + 10% Fetal Bovine Serum (Integro, Zaandam, the Netherlands) with serial dilutions of UCARTCS1 cells or control cells, both labeled with Violet Tracer (Invitrogen), in 96-well U-bottom plates for 24 hours at 37^◦^C. The mean percentage of viable MM cells within BM-MNCs was 14.63% (range 0.36–62) at baseline and 15.43% (range 0.30–71) after 24 hours in the untreated conditions.

The survival of primary CD138^+^ MM cells in the BM-MNCs was determined by flow cytometry, as previously described.^25^,^28^ Briefly, surviving MM cells were enumerated by single platform flow cytometric analysis of CD138^+^ cells in the presence of Flow-Count Fluorospheres (Beckman Coulter) and LIVE/DEAD Fixable Dead Cell Stain Near-IR fluorescent reactive dye (Invitrogen). Percentage of cell lysis induced by UCARTCS1 was calculated using the following formula: % lysis cells=1−(absolute number of surviving cells in treated wells/ absolute number of surviving cells in untreated wells)×100%.^27^

### Cytokine measurements

To determine cytokine and granzyme B production by UCARTCS1 and control non-transduced T-cells, cell supernatants were collected after 24-hour cytotoxicity assays. Cytokine production was measured by flow cytometry using the Cytometric Bead Array Human Th1/Th2/Th17 Cytokine Kit (BD Biosciences). Granzyme B was measured by ELISA using an ELISA development kit (Mabtech AB). Assays were performed according to the manufacturer’s protocol.

### Xenograft models

Animal studies were performed in compliance with international OECD principles on GLP, fully recognized by worldwide Regulatory Authorities (FDA, EMA and PMDA) at Accelera SRL (Italy), whose animal facilities have full accreditation by AAALAC International (Association for Assessment and Accreditation of Laboratory Animal Care).

NOD SCID Gamma female mice (NOD.Cg-*Prkdc^scid^ Il2rg^tm1Wjl^*/SzJ mice, also named NSG mice) were obtained from Charles River Italy, 6 weeks old. Mice were injected IV on day −10 with 5×10^6^ luciferase (LUC) and green fluorescent protein (GFP)-expressing MM.1S cells. 10 days later, mice were IV injected with 3×10^6^ UCARTCS1 cells, 10×10^6^ UCARTCS1 cells, or with vehicle (PBS). MM tumor burden was monitored with bioluminescence on days −1, day 14, day 21, day 28, and day 35. Overall survival was also evaluated. Four or five mice per treatment group were sacrificed on day 15 in order to assess the presence of UCARTCS1 cells and MM.1S cells in blood, BM and spleen.

### Statistics

Comparisons between variables were performed using two-tailed (paired) Student’s t-test or analysis of variance in case data followed a normal distribution and with Kruskal-Wallis, Mann-Whitney test or Wilcoxon matched-pairs signed-rank test in case the data did not follow a normal distribution.

To assess the relationship between patient and tumor characteristics and response to UCARTCS1, samples were divided into two groups based on the median value of each variable (except for cytogenetic risk status and for gain/amp 1q status).

For in vivo studies, logrank test was used to determine differences in survival between groups. Statistical analyses were performed in GraphPad Prism (V.9). P values below 0.05 were considered significant.

## Results

### Generation of universal CAR T-cells targeting SLAMF7

**Figure 1:**
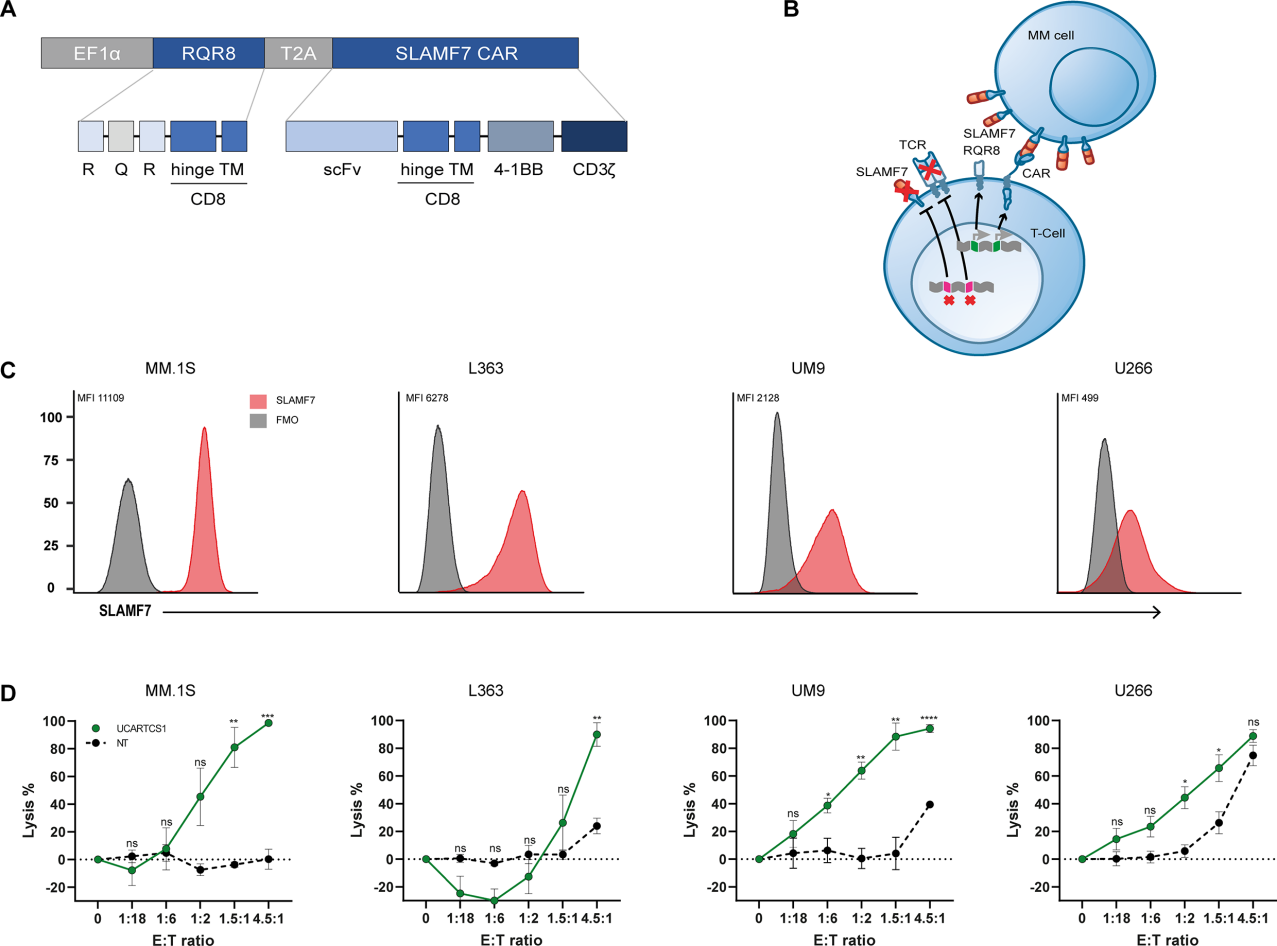
UCARTCS1-mediated lysis of cell lines in vitro. (A) Schematic representation of UCARTCS1 CAR construct containing scFv, CD8-derived hinge and transmembrane domain, 4-1BB, and CD3ζ signaling domains. The CAR is coexpressed with RQR8 as safety feature, by conferring sensitivity to rituximab. (B) UCARTCS1 cell expressing SLAMF7-targeting CAR and RQR8. The TCRαβ receptor is disrupted by knocking out the TRAC gene. Fratricide is prevented by SLAMF7 gene knockout. (C) Flow cytometry histogram overlays depicting SLAMF7 cell surface expression on four MM cell lines (MM.1S, L363, UM9 and U266; red histogram), compared with FMO (fluorescence minus one; gray histogram). SLAMF7 MFI is provided. (D) UCARTCS1-mediated lysis in four MM cell lines. Non-transduced (NT), SLAMF7/TCRαβ double knock-out T-cells were used as control. MM cell lysis was assessed using a 24-hour BLI-based cytotoxicity assay. Data represent mean MM cell lysis±SEM of three independent experiments performed in duplicate. Unpaired t-test was used to calculate significance between both groups. *p<0.05, **p<0.01, ***p<0.001, ****p<0.0001. E:T ratio, effector to target-ratio; MM, multiple myeloma; MFI, median fluorescence intensity; ns, not significant; scFv, single-chain variable fragment; TCR, T-cell receptor

UCARTCS1 cells were produced from T-cells obtained from healthy donors. The CAR construct consist of four components: a single-chain variable fragment (scFv) targeting SLAMF7, a CD8α-derived hinge and transmembrane domain, a costimulatory domain (4-1BB) and a CD3ζ signaling domain. A lentiviral vector was used for the expression of the SLAMF7 CAR, which was coexpressed with RQR8, an epitope-based marker/suicide gene that confers sensitivity to the anti-CD20 antibody rituximab (figure 1A,B). The genes coding for the TCRα chain of the TCRαβ receptor and for SLAMF7 were disrupted using TALEN gene-editing technology. These modifications aim to minimize the potential risk for allo-TCR-mediated GvHD (eliminating TCR-mediated recognition of histocompatibility antigens that can lead to GvHD) and diminish SLAMF7-mediated fratricide. Evaluation of potential TALEN mediated off-target cleavage activity was performed, showing absence of cleavage in candidate sites identified using an unbiased genome-wide approach. Karyotyping analysis and FISH were performed to detect translocations. The only clonal aberration observed included the expected genomic rearrangements (balanced translocations) involving the TRAC/SLAMF7 cleavage sites, although at a low frequency (see online supplemental methods for further details). Healthy donor T-cells displayed a mostly naïve and central memory phenotype on day 1 before UCARTCS1 production and exhibited a mixed effector memory and central memory phenotype in both CD4^+^ and CD8^+^ T-cell subsets after UCARTCS1 production (online supplemental figure 2C,D).

### Fratricide of CD8^+^ T-cells is prevented by SLAMF7 gene inactivation

Because SLAMF7 is predominantly expressed in CD8^+^ T-cells (online supplemental figure 3A), SLAMF7-mediated fratricide may result in a product with a low proportion of CD8^+^ T-cells. We therefore investigated the impact of SLAMF7 deletion on the composition of the CAR T-cell product. To this end, we transduced the SLAMF7 CAR in SLAMF7 knockout T-cells or non-edited T-cells. SLAMF7 gene-editing allowed to maintain adequate levels of CD8^+^ T-cells, compared with what was observed in non-edited cells (proportion of CD8^+^ T-cells: 53.8% vs 10.3%, p<0.0001, (online supplemental figure 3B). As expected, in the absence of SLAMF7-mediated fratricide, the proportion of CD8^+^ T-cells was similar in non-transduced T-cells (no CAR) with or without SLAMF7 gene-editing. Because SLAMF7 knockout may lead to less antigenic stimulation during the manufacturing process, we also evaluated the impact of SLAMF7 gene-editing on T-cell differentiation. There was a trend for SLAMF7 knockout CAR T-cells to be less differentiated, compared with non-edited CAR T-cells (online supplemental figure 3C). Next, we evaluated the cytotoxic capacity of SLAMF7-targeting CAR T-cells with or without SLAMF7 gene-editing in an in vitro cytotoxicity assay. Importantly, there was no significant difference in CAR-mediated MM cell lysis between SLAMF7 knockout CAR T-cells or non-edited CAR T-cells (online supplemental figure 3D).

### Activity of UCARTCS1 against MM cell lines

We first evaluated the activity of UCARTCS1 against four different MM cell lines (MM.1S, L363, UM9, U266) with different SLAMF7 expression levels (figure 1C). MM cell lines were incubated with UCARTCS1 cells or control (non-transduced, SLAMF7/TCRαβ double knockout) T-cells at different E:T ratios for 24 hours. UCARTCS1 induced potent dose-dependent lysis in all 4 cell lines (figure 1D). Extent of UCARTCS1-mediated cytotoxity was not clearly associated with SLAMF7 expression levels. The cytotoxic activity of UCARTCS1 was significantly better than that of the control (non-transduced, SLAMF7/TCRαβ double knockout) T-cells, indicating the CAR-dependency of the lysis by UCARTCS1 cells. Control T-cells did not induce killing of MM cells, except at the highest E:T ratio in two out of four MM cell lines (UM9 and U266).

### UCARTCS1 effectively kills primary MM cells obtained from newly diagnosed and relapsed/refractory patients

To evaluate the efficacy of UCARTCS1 cells against primary MM cells obtained from both NDMM and RRMM patients (see table 1 for patient characteristics), we treated BM-MNC (containing tumor cells, as well as autologous effector cells, and immune suppressor cells) from 29 patients with UCARTCS1 cells or control T-cells for 24 hours in flow cytometry based cytotoxicity assays.

**Table 1:** Characteristics of MM patients

	NDMM (n=10)	RRMM (n=10)	DARA-R MM (n=9)
Age, years, median (range)	61 (51–86)	63 (54–77)	65 (56–80)
Male, n (%)	9 (90)	6 (60)	6 (67)
LDH, U/L			
Median (range)	203 (133–334)	185 (58–1505)	200 (81–294)
Unknown, n (%)	3 (30)	0	0
Elevated, n (%)	1 (14)	1 (10)	2 (22)
eGFR, mL/min/1.73 m^2^			
Median (range)	88 (12–90)	86 (39–90)	82 (45–90)
Unknown, n (%)	1 (10)	0	0
B_2_-microglobulin, mg/L			
Median (range)	3,8 (2,1–12)	NA	NA
Unknown, n (%)	3 (30)		
M protein, n (%)			
IgG	3 (33)	6 (60)	3 (33)
IgA	2 (22)	2 (20)	5 (56)
FLC only	4 (44)	2 (20)	1 (11)
Non-secretor	0	0	0
Unknown	1 (10)	0	0
ISS score at diagnosis, n (%)			
1	3 (33)	NA	NA
2	3 (33)		
3	3 (33)		
Unknown	1 (10)		
High risk cytogenetics,* n (%)	2 (22)	1 (11)	6 (67)
del(17 p)	2	0	3
t(4;14)	1	1	3
t(14;16)	0	0	0
Unknown	1	1	0
Prior lines, median (range)	0	3 (2–5)	4 (1–6)
Prior autologous SCT, n (%)	NA	7 (70)	8 (89)
Single		4 (40)	6 (67)
Double		3 (30)	2 (22)
Prior allogeneic SCT, n (%)	NA	0	1 (11)
Thalidomide, n (%)	NA		
Exposed		4 (40)	2 (22)
Refractory†		0	0
Lenalidomide, n (%)	NA		
Exposed		9 (90)	8 (89)
Refractory		8 (80)	7 (78)
Pomalidomide, n (%)	NA		
Exposed		4 (40)	0
Refractory		4 (40)	0
Bortezomib, n (%)	NA		
Exposed		9 (90)	9 (100)
Refractory		2 (20)	0
Carfilzomib, n (%)	NA		
Exposed		3 (30)	2 (22)
Refractory		2 (20)	1 (11)
Ixazomib, n (%)	NA		
Exposed		1 (10)	2 (22)
Refractory		1 (10)	2 (22)
Daratumumab, n (%)	NA		
Exposed		0	9 (100)
Refractory		0	9
Nivolumab, n (%)	NA		
Exposed		0	5 (56)
Refractory		0	5 (56)
IMiD,‡ n (%)	NA		
Exposed		10 (100)	9 (100)
Refractory		10 (100)	7 (78)
PI,§ n (%)	NA		
Exposed		10 (100)	9 (100)
Refractory		5 (50)	3 (33)
IMID and PI, n (%)	NA		
Exposed		10 (100)	9 (100)
Refractory		5 (50)	3 (33)
Triple class,¶ n (%)	NA		
Exposed		0	9 (100)
Refractory		0	3 (33)
Radiotherapy, n (%)			
No	8 (89)	5 (50)	7 (78)
Yes	1 (11)	5 (50)	2 (22)
Unknown	1 (10)	0	0
Extramedullary plasmacytomas, n (%)			
No	9 (100)	8 (80)	9 (100)
Yes	0	2 (20)	0
Unknown	1 (10)	0	0

* Based on the presence of del(17p p), t(4;14) and/or t(14;16).

† Refractory disease was defined as progressive disease during therapy, no response (less than partial response), or progressive disease within 60 days of stopping treatment, according to the International Uniform Response Criteria for MM. BM samples were obtained at the time of diagnosis for newly diagnosed patients, and at the time of progression for other patients (n=19). Of these 19 patients, 8 progressed during their last line of therapy, and 7 patients progressed within 60 days after treatment was stopped. Four samples were collected at progression >60 days after stopping the last line of treatment.

‡ Thalidomide, lenalidomide and/or pomalidomide.

§ Bortezomib, carfilzomib, and/or ixazomib.

¶ At least 1 IMiD, 1 PI, and CD38-targeting antibody.

BMbone marrowDRMMdaratumumab refractory MMeGFRestimated glomerular filtration rateFLCfree light chainsIMiDimmunomodulatory drugISSInternational Staging SystemLDHlactate dehydrogenaseMMmultiple myelomaNAnot applicableNDMMnewly diagnosed MMPIproteasome inhibitor RRMMrelapsed/refractory MMSCTstem cell transplantation

**Figure 2:**
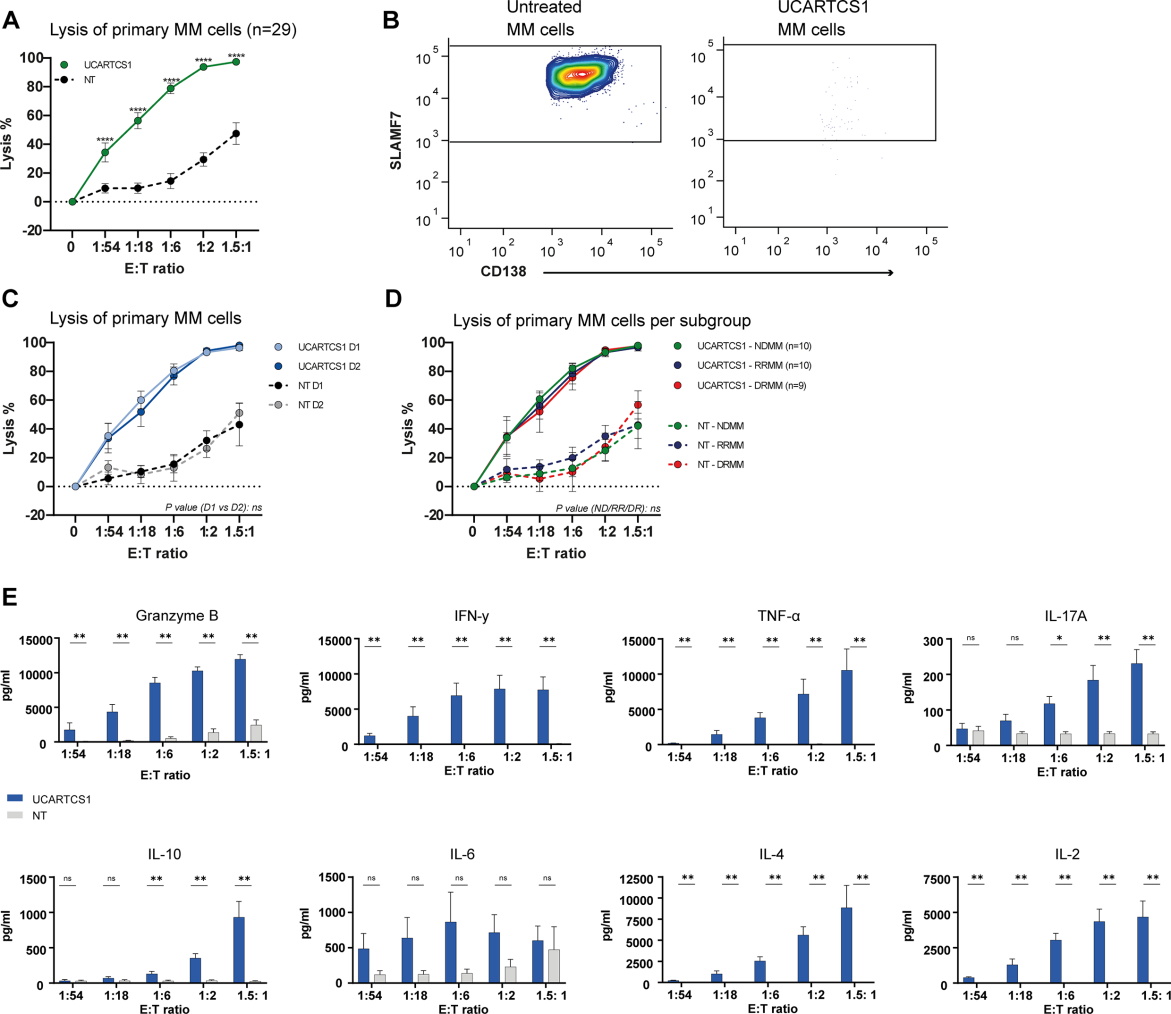
UCARTCS1-mediated lysis of primary MM cells. (A) BM-MNC obtained from 29 patients were incubated with UCARTCS1 cells or control (non-transduced (NT), SLAMF7/TCRαβ double knock-out) T-cells at different E:T ratios for 24 hours, after which the surviving MM cells were enumerated using flow cytometric analysis. Data represent mean±SEM. Mann-Whitney U test was used to calculate significance between both groups. (B) Representative flow cytometry contour plots showing SLAMF7-positive MM cells from a newly diagnosed MM patient, untreated (left) or treated with UCARTCS1 cells (right; E:T ratio of 1.5:1). After exclusion of doublets and dead Fixable Near-IR positive cells, MM cells were identified as CD38^+^ CD138^+^ cells. (C) UCARTCS1-mediated lysis of MM cells was similar when using UCARTCS1 cells from two different healthy T-cell donors. UCARTCS1 cells (light blue) and control NT T-cells (black) from donor one were tested with 16 BM samples; UCARTCS1 cells (dark blue) and control NT T-cells (gray) from donor two were tested with 13 BM samples. Mann-Whitney test was used to evaluate significance between both groups. (D) UCARTCS1-mediated lysis of MM cells in patient subgroups according to prior treatment: 10 BM samples from newly diagnosed MM patients (NDMM; green), 10 from daratumumab-naïve RRMM patients (RRMM; blue), and nine from daratumumab-refractory MM patients (DRMM; red). Solid lines represent UCARTCS1 cells and dotted lines control (non-transduced (NT), SLAMF7/TCRαβ double knock-out) T-cells. Data represent mean±SEM. Patient subgroups were compared using the Kruskal-Wallis test. (E) IFN-y, TNF-α, IL-17a, IL-10, IL-6, IL-4, IL-2, and granzyme B were measured in the cell supernatants of BM-MNCs treated with UCARTCS1 (blue) or control (non-transduced (NT), SLAMF7/TCRαβ double knock-out) T-cells (gray) for 24 hours by using a flow cytometry based assay (cytokines) or an ELISA (granzyme B). BM-MNCs were obtained from six DRMM patients. Data represent mean±SEM. Mann-Whitney U test was used to calculate significance between both groups. *p<0.05, **p<0.01, ****p<0.0001. BM-MNCs, bone marrow mononuclear cells; E:T ratio, effector to target-ratio; MM, multiple myeloma; ns, not significant; TCR, T-cell receptor.

UCARTCS1 cells effectively lysed primary MM cells from all 29 patients in a dose-dependent way, with a mean maximal lysis of 97.3% (figure 2A,B). The control T-cells showed significantly lower anti-MM activity in these patient samples, confirming the requirement of CAR expression for efficient lysis. UCARTCS1 cells used in these ex vivo experiments were obtained from two healthy donors and no significant difference in efficacy was observed between the UCARTCS1 cells derived from the different donors (figure 2C). Importantly, there was no difference in UCARTCS1-mediated MM cell lysis between newly diagnosed patients (n=10), daratumumab-naïve relapsed/refractory patients (n=10; median of 3 prior therapies, 100% IMiD-refractory, 50% PI-refractory) and daratumumab-refractory patients (n=9; median of 4 prior therapies, 78% IMiD-refractory, 33% PI-refractory) (figure 2D). This indicates that exposure and resistance to prior therapies did not result in reduced susceptibility to UCARTCS1-mediated lysis ex vivo. Next, we evaluated cytokines and granzyme B levels in the supernatants of UCARTCS1 treated BM samples obtained from DRMM patients. UCARTCS1-mediated MM cell lysis was accompanied by a dose-dependent increase in the secretion of granzyme B and cytokines (IFNy, TNF-α, IL17a, IL-10, IL-6, IL-4 and IL-2). Control T-cells produced significantly lower levels of all cytokines and released significantly less granzyme B at all E:T ratios tested (figure 2E).

### Impact of tumor and patient characteristics on UCARTCS1 activity

**Figure 3:**
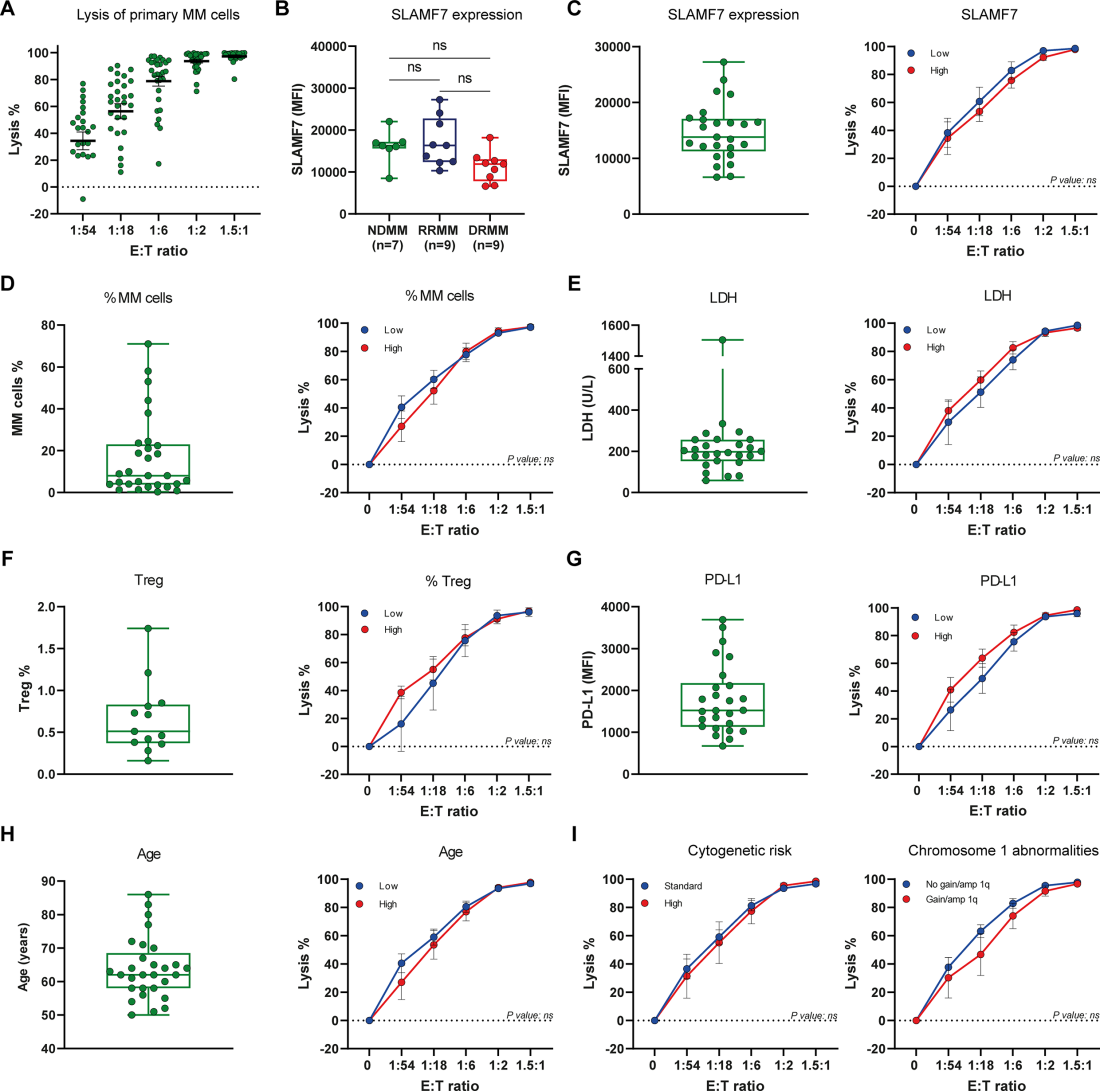
Impact of tumor and patient characteristics on UCARTCS1 activity. (A) Shown are the individual UCARTCS1-mediated MM cell lysis values of all 29 MM patients (10 newly diagnosed MM patients (NDMM), 10 daratumumab-naïve RRMM patients (RRMM), and 9 daratumumab-refractory MM patients (DRMM)). In these experiments, BM-MNCs were incubated with UCARTCS1 cells at different E:T ratios for 24 hours, after which the surviving MM cells were enumerated using flow cytometric analysis. Data represent mean±SEM. Each dot represents an individual BM sample. (B) SLAMF7 expression level on MM cells from 7 NDMM, 9 RRMM and 9 DRMM patients, as determined by flow cytometry. Each dot represents an individual sample, with box and whiskers, representing median, 25th–75th percentile, and range. Groups were compared with Kruskal-Wallis test. (C–I) The impact of tumor and patient characteristics on MM cell lysis was assessed by constructing dose-response curves for UCARTCS1-mediated MM cell lysis, according to median SLAMF7 expression level (n=25), median frequency of MM cells (n=29), median LDH level (n=26), median frequency of Tregs (n=13), median PD-L1 expression level (n=26), median age (n=29), cytogenetic risk status (n=27), and presence/absence of chromosome one abnormalities (gain/amp 1q) (n=27). Data represent mean±SEM. (C–I) Blue lines represent samples with a value below or equal to the median value, and red lines samples above the median value. Groups were compared using Mann-Whitney U test. (C–H) Each dot represents an individual sample, with box and whiskers, representing median, 25th–75th percentile, and range. Amp 1q, amplification 1q; BM-MNCs, bone marrow mononuclear cells; E:T ratio, effector to target-ratio; LDH, lactate dehydrogenase; PD-L1, programmed death-ligand 1; RRMM, relapsed/refractory multiple myeloma; TCR, T-cell receptor; Treg, regulatory T-cell; ns, not significant.

Although all 29 patients responded to UCARTCS1 treatment in our ex vivo assays, there was variability in sensitivity at the lower E:T ratios (figure 3A). Since prior therapy did not explain the heterogeneity in response, we examined whether differences in baseline tumor features and immune cell composition have an impact on the susceptibility of MM cells toward UCARTCS1.

We first examined the relationship between tumor SLAMF7 expression levels and UCARTCS1-mediated lysis in 25 out of 29 patients from whom enough BM material was collected (figure 3B,C). Despite heterogeneous interindividual SLAMF7 levels, there was no significant difference in SLAMF7 expression levels between newly diagnosed or relapsed/refractory patients including daratumumab-refractory patients, indicating that SLAMF7 expression was not affected by previous therapy or disease progression. We also did not identify SLAMF7-negative subpopulations in any of these patients. To explore the impact of SLAMF7 expression on response, patients were divided into two groups according to whether SLAMF7 expression was above or below the median value of all tested samples, and dose-response curves were compared between both groups. There was no difference in UCARTCS1 activity between samples with high or low SLAMF7 cell surface expression (figure 3C). Dose-response curves show that UCARTCS1 activity was also independent of MM cell frequency, serum LDH level, Treg counts, PD-L1 expression levels on tumor cells, and patients’ age (figure 3D–H). Importantly, the presence of high-risk cytogenetic abnormalities (del(17 p), t(4; 14) and/or t(14; 16)) did not impair UCARTCS1 activity. In addition, UCARTCS1 activity was not affected by presence of gain/ampl 1q (figure 3I).

### Effect of UCARTCS1 on non-malignant immune cells

**Figure 4:**
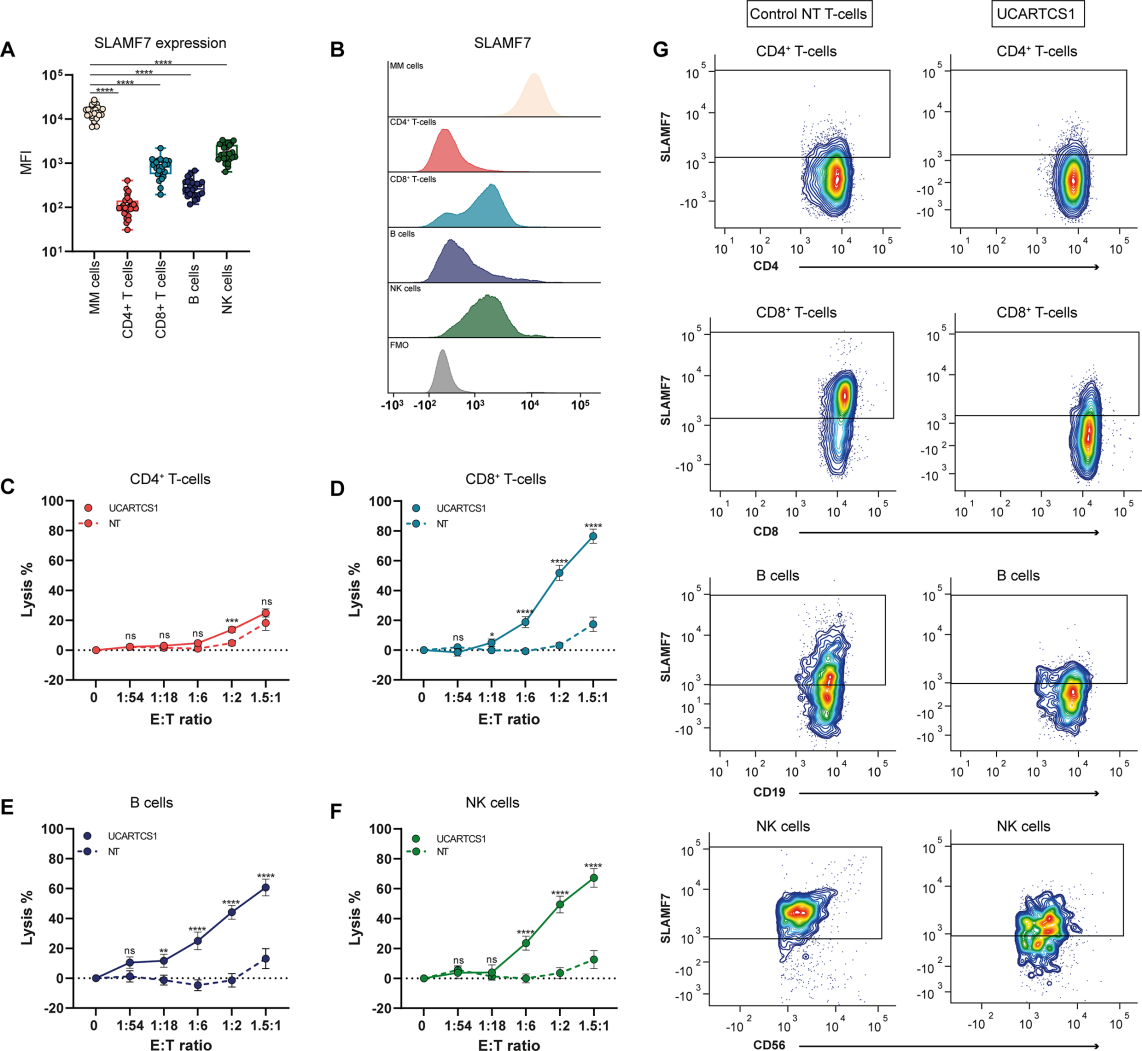
Effect of UCARTCS1 on non-malignant immune cells. (A) SLAMF7 expression on MM cells was compared with SLAMF7 expression on CD4^+^ T-cells, CD8^+^ T-cells, B cells and NK cells in 25 BM samples from MM patients (the same patients’ BM samples were also used in killing assays) by Wilcoxon matched-pairs test. Each dot represents an individual sample, with box and whiskers, representing median, 25th–75th percentile, and range. (B) Representative flow cytometry histogram overlays depicting SLAMF7 expression on MM cells, CD4^+^ T-cells, CD8^+^ T-cells, B cells and NK cells in a BM sample from a newly diagnosed MM patient. Fluorescence minus one (FMO) is depicted in gray (gated on MM cells). (C–F) BM-MNCs obtained from 29 MM patients were incubated with UCARTCS1 cells or control (non-transduced (NT), SLAMF7/TCRαβ double knock-out) T-cells at different E:T ratios for 24 hours after which surviving CD4^+^ T-cells, CD8^+^ T-cells, B cells and NK cells were enumerated using flow cytometric analysis. Solid lines represent UCARTCS1 cells and dotted lines control NT T-cells. Data represent mean±SEM. Groups were compared using Mann-Whitney U test. (G) Representative flow cytometry contour plots showing CD4^+^ T-cells, CD8^+^ T-cells, B cells and NK cells from a newly diagnosed MM patient after treatment with control (non-transduced (NT), SLAMF7/TCRαβ double knock-out) T-cells (left) or UCARTCS1 cells (right) (E:T ratio of 1.5 : 1). *p<0.05, **p<0.01, ***p<0.001, ****p<0.0001. BM-MNCs, bone marrow mononuclear cells; E:T ratio, effector to target-ratio; MM, multiple myeloma; ns, not significant; TCR, T-cell receptor.

Because SLAMF7 is not only expressed in MM cells, but also in other normal immune cells, we assessed the expression level of SLAMF7 on various immune cell subsets in the BM samples from 25 MM patients, from whom enough material was available (figure 4A,B). SLAMF7 was also expressed in CD4^+^ T-cells (median MFI: 99), CD8^+^ T-cells (median MFI: 824), B cells (median MFI: 263), and NK cells (median MFI: 1593), but cell surface expression was significantly lower compared with that observed on MM cells (figure 4A).

We next evaluated the potential cytotoxic activity of UCARTCS1 against these healthy immune cells. To this end, BM-MNCs from 29 MM patients (same as described above) were incubated with UCARTCS1 cells or control T-cells in 24 hours flow cytometry-based cytotoxicity assays. Only at the higher dose levels, UCARTCS1 treatment eliminated SLAMF7^+^ non-malignant immune cells while sparing the SLAMF7^low^ fraction in each immune cell subset. Importantly, lysis of normal cells was less pronounced than MM cell lysis. In these experiments, CD4^+^ T-cells were the least sensitive to UCARTCS1, which can be explained by their low SLAMF7 expression, compared with CD8^+^ T-cells, NK cells or B cells (figure 4C–G).

We also evaluated the activity of UCARTCS1 against CD4^+^ Tregs. SLAMF7 expression was slightly lower on Tregs (median MFI: 67), compared with CD4^+^ non-Treg T-cells (Median MFI: 112; online supplemental figure 4A). However, in both Tregs and CD4^+^ non-Treg cells, UCARTCS1 did not induce more lysis than what was observed with control T-cells (online supplemental figure 4B,C).

### In vivo activity of UCARTCS1

**Figure 5:**
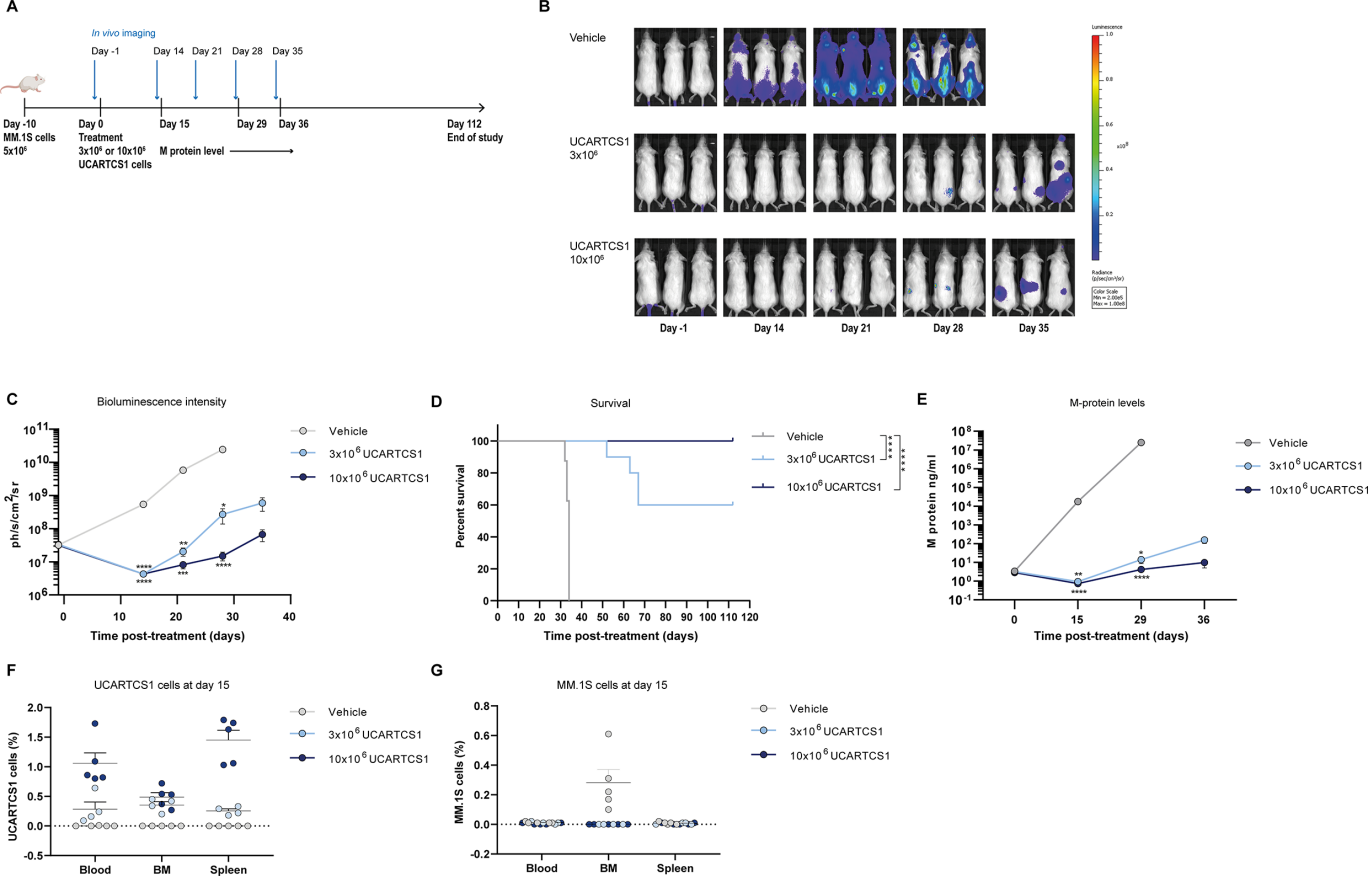
In vivo activity of UCARTCS1. (A) NSG mice injected with LUC-GFP-transduced MM.1S (iv) on day −10 and treated with a single dose of 3×10^6^ UCARTCS1 cells, or 10×10^6^ UCARTCS1 cells, or vehicle control (on day 0). (B, C) BLI readout performed on day −1, day 14, day 21, day 28, and day 35 to evaluate tumor progression in the different treatment groups. Both treatment groups were compared with vehicle control using Kruskal Wallis test. (D) Survival curves of mice treated with a single dose of 3×10^6^ UCARTCS1 cells, a single dose of 10×10^6^ UCARTCS1 cells, or vehicle control. Groups were compared using logrank test. (E) M-protein levels (ng/mL) assessed in mice at day 15, day 29 and day 36. Both treatment groups were compared with vehicle control using Kruskal-Wallis test. Data represent mean±SEM. (F, G) Shown are percentages of UCARTCS1 cells and MM.1S cells in the blood, BM and spleen of the subset of mice sacrificed at day 15 (4–5 mice per group). Presence of UCARTCS1 and MM.1S cells was assessed using flow cytometric analysis. UCARTCS1 cells were identified by the expression of human CD45 (hCD45) and by negativity for GFP. MM.1S-LUC-GFP tumor cells were identified as GFP^+^/hCD45^-^ cells. Data represent mean±SEM. Each dot represents an individual mouse. *p<0.05, **p<0.01, ***p<0.001, ****p<0.0001. BLI, bioluminescence imaging; LUC, luciferase; MM, multiple myeloma; ns, not significant; NSG, NOD SCID gamma mice.

The in vivo activity of UCARTCS1 was evaluated in immunodeficient NSG mice engrafted with MM.1S cells with monitoring of tumor burden by BLI. Mice were treated with a single dose of 3×10^6^ or 10×10^6^ UCARTCS1 cells (large-scale batch) or with PBS as control (figure 5A). From day 14, we observed increased BLI signal, indicating disease progression in the mice of the control group, compared with mice treated with UCARTCS1 cells (figure 5B,C). In addition, UCARTCS1 cells significantly improved overall survival of mice (figure 5D). In line with these results, M-protein levels in mice treated with UCARTCS1 cells were significantly lower than in control mice (figure 5E). The antitumor response was dose-dependent with superior response in mice treated with 10×10^6^ UCARTCS1 cells (figure 5C–E). We also assessed the presence of UCARTCS1 cells at day 15 postinfusion in blood, BM and spleen in a subset of mice. The percentage of UCARTCS1 cells correlated with the administered dose, with the highest percentage of UCARTCS1 cells observed in mice treated with 10×10^6^ UCARTCS1 cells (figure 5F). MM.1S cells were not detected in blood, BM, or spleen of UCARTCS1-treated mice at day 15 while tumor cells were present in the BM of control mice (figure 5G). All mice treated with 10×10^6^ UCARTCS1 cells (not sacrificed at day 15; n=10) were still alive at the end of study (day 112). At that time, UCARTCS1 cells were detectable at low levels in blood (median: 0.015%), BM (median: 0.035%) and spleen (median: 0.035%). MM.1S cells were detected in blood in only 2 of 10 mice (frequency of 0.01% in both mice), in BM in 1 mouse (0.01%), and spleen in 1 mouse (0.03%) (online supplemental figure 5A,B).

## Discussion

We here show that UCARTCS1 cells, generated from healthy donor T-cells, have substantial activity against MM cell lines and primary MM cells from patients with both newly diagnosed disease or heavily pretreated MM. UCARTCS1 cells also showed efficacy in an MM mouse model. Altogether these results support the potential benefits of UCARTCS1 in MM patients.

BCMA-targeted therapies, including antibody-drug conjugates, autologous CAR T-cell and T-cell engagers, are increasingly used because of their high activity, but relapses continue to be observed. Mechanisms underlying resistance include BCMA target loss resulting from deletions or mutational events.^7^,^11^ Antigen escape is also a frequent cause of acquired resistance to GPRC5D-targeted immunotherapies.^12 29 30^ Altogether this underscores the need for T-cell immunotherapies that target alternative tumor-associated antigens. UCARTCS1 is the first SLAMF7-targeting allogeneic CAR T-cell product evaluated in patients.^31^ Importantly, we here show that SLAMF7 continues to be expressed in heavily pretreated MM patients, indicating that treatment with IMiDs, proteasome inhibitors or CD38 antibodies does not impact SLAMF7 expression on MM cells. Preserved SLAMF7 expression, and a different mode of action without cross-resistance to other anti-MM agents, may explain why UCARTCS1 cells had comparable efficacy against primary MM cells in samples from newly diagnosed and heavily pretreated, triple-class refractory MM patients. Another possible advantage of allogeneic CAR T-cells obtained from healthy donors is their potentially higher activity, when compared with autologous CAR T-cells prepared from patient-derived T-cells, which frequently have functional defects due to cumulative exposure to anti-MM agents.^32^

MM is characterized by an immunosuppressive microenvironment with a high frequency of immune suppressor cells such as Tregs. We and others have recently shown that activity of T-cell redirecting antibodies can be reduced by Tregs.^33^,^35^ Although Treg expansion has been described in patients with MM without response to BCMA CAR T-cell therapy,^36^ the precise impact of Tregs on CAR T-cell activity remains unclear. In our ex vivo assays, we used MNCs isolated from BM aspirates from MM patients, which not only contain MM cells but also stromal cells and immune suppressor cells. Importantly, the extent of spontaneous MM cell death is minimal in our short-term flow cytometry-based cytotoxicity experiments using whole BM-MNCs. UCARTCS1 killed MM cells regardless of the presence of high Treg counts. Also, the presence of a large proportion of tumor cells, high PD-L1 levels on tumor cells or high-risk cytogenetic abnormalities did not result in reduced susceptibility to UCARTCS1-mediated lysis. Still, additional studies are necessary to better evaluate the impact of these parameters on UCARTCS1 therapy because limitations of our ex vivo killing assay include the inability to study long-term effects such as CAR T-cell exhaustion and persistence. Furthermore, additional studies are needed to clarify the impact of other immune suppressor cells in the tumor microenvironment, such as regulatory B cells and myeloid-derived suppressor cells, on CAR T-cell function. In some studies, inferior results were obtained with immunotherapy in elderly patients, which can be explained by an age-related decline of the immune system.^33 37^ However, because UCARTCS1 cells are generated from relatively young healthy donors, there was no negative impact of patients’ age on efficacy of UCARTCS1 in our ex vivo killing assays.

We also evaluated the potential of UCARTCS1 to induce on-target/off-tumor toxicity. Expectedly, our data show that UCARTCS1 kills a subset of B cells, NK cells, and T-cells with highest levels of SLAMF7, while sparing cells with low or no SLAMF7 expression. The impact of UCARTCS1 on CD8^+^ T-cells was more pronounced than on CD4^+^ T-cells, which is likely related to the lower SLAMF7 expression on CD4^+^ T-cells. These results are consistent with our data showing fratricide of CD8^+^ T-cells during the CAR T-cell manufacturing process when SLAMF7 was not genetically deleted. Indeed, SLAMF7 knockout was crucial to prevent CD8^+^ T-cell fratricide during the production process, resulting in an improved CD4^+^/CD8^+^ T-cell ratio in the final CAR T-cell product. Additionally, editing the SLAMF7 gene may also lead to the production of less differentiated cells, as T-cells undergo less antigenic stimulation during the manufacturing process. Altogether, this could improve fitness and persistence of UCARTCS1 cells.^7 38 39^ We also show that SLAMF7 gene-editing did not affect the cytotoxic capacity of SLAMF7-targeting CAR T-cells, which is in agreement with a previous study showing that SLAMF7 was not required for CAR T-cell efficacy in an MM mouse model.^21^

The differential impact of UCARTCS1 on normal immune cells is also in accordance with a previous preclinical study which showed a more prominent impact of autologous SLAMF7-targeting CAR T-cells on CD8^+^ T-cells versus CD4^+^ T-cells.^16^ However, the spared SLAMF7^−/low^ fraction in each T-cell subset contained sufficient functional lymphocytes, including virus-specific T-cells.^16^ Thus despite the reduction, especially of CD8^+^ T-cell counts, the remaining population of SLAMF7^−/low^ T-cells should be sufficient to confer protective immunity against pathogens. On the other hand, evaluation of UCARTCS1 in humans should provide a better understanding if, and to what extent, UCARTCS1 could induce immune deficiency and increased risk of infections. UCARTCS1 also contains a mimotope that confers susceptibility to the anti-CD20 antibody rituximab, enabling the elimination of CAR T-cells in case of severe toxicity.^40^

Interestingly, it was recently reported that BM-resident cancer associated fibroblasts (CAFs) also express SLAMF7(41). Because CAFs inhibit CAR T-cell antitumor activity and promote MM progression, simultaneously targeting both CAFs and MM cells with UCARTCS1 may reverse tumor microenvironment-induced immunosuppression and enhance antitumor activity of CAR T-cells.^41^

Autologous CAR T-cell products targeting SLAMF7 are also being explored in MM.^616 20^,^22^ However, clinical data have to the best of our knowledge not yet been reported. UCARTCS1 is an off-the-shelf allogeneic CAR T-cell product, which can be directly administered to MM patients without the need to wait for a time-consuming manufacturing process, which can be as long as 6–8 weeks for CAR T-cell products that are generated from the patient’s own T-cells. Indeed, several studies have shown that a proportion of patients who underwent leukapheresis never receive autologous CAR T-cells because of disease progression, clinical deterioration during manufacturing, or manufacturing failure (ide-cel: 8.6%–11.4%; cilta-cel: 14.2%–15.4%).^3 4 42 43^ Allogeneic off-the-shelf CAR T-cells manufactured using T-cells from healthy donors may help to overcome these barriers. Manufacturing of patient-specific autologous CAR T-cells is not only a lengthy process but also very costly.^6^ Large-scale manufacturing of allogeneic CAR T-cells from healthy donors, whereby cells from one donor can be used to treat a large number of patients, has the potential to lower the costs associated with manufacturing and lead to a wider application and availability of CAR T-cell therapy. Nevertheless, it is anticipated that UCARTCS1 cells will be rejected by the immune system of the patient following recovery from the lymphodepleting preconditioning regimen, due to the allogeneic nature of these cells. Although this will limit the persistence of CAR T-cells, there is a therapeutic window in which UCARTCS1 cells could display potent antitumor activity. Furthermore, off-the-shelf availability provides the possibility to perform redosing, if needed. Proof of concept of UCARTCS1 activity has been reported in preliminary data from the phase 1 MELANI-01 trial (NCT04142619), showing early antitumor activity in heavily pretreated patients with RRMM.^31^ Although UCARTCS1 cell expansion was associated with clinically meaningful responses, this was accompanied by notable toxicity including grade 2–4 cytokine release syndrome (CRS) and grade 5 events. Besides CRS, other UCARTCS1-associated adverse events included hemophagocytic lymphohistiocytosis, lymphopenia, and infections (including disseminated mucormycosis, and pseudomonal pneumonia).^31^ In addition, initial results from the phase 1 UNIVERSAL study with the first allogeneic BCMA-targeted CAR T-cell therapy (ALLO-715), also support the feasibility, efficacy, and safety of allogeneic CAR T-cell therapy for patients with RRMM.^44 45^

In conclusion, SLAMF7 is an attractive target for T-cell immunotherapies due to its high and consistent expression on MM cells, with expression in normal tissues restricted to the hematopoietic system including subsets of T-cells, B cells and NK cells. UCARTCS1 demonstrated marked anti-MM activity both against MM cell lines and primary MM cells, as well as in an MM mouse model. In our ex vivo experiments we showed that the extent of prior treatment did not have an impact on the susceptibility of MM cells toward UCARTCS1. Overall, these preclinical data support the evaluation of UCARTCS1 in patients with advanced MM.

## supplementary material

10.1136/jitc-2023-008769 online supplemental file 1

## Data Availability

Data are available on reasonable request.
