# Supplementary material for: Preclinical activity of allogeneic SLAMF7-specific CAR T-cells (UCARTCS1) in multiple myeloma
Source: J Immunother Cancer. 2024 Jul 25;12(7):e008769. doi: 10.1136/jitc-2023-008769 (PMC11284884; doi:10.1136/jitc-2023-008769)
Supplement: online supplemental file 1 [file jitc-12-7-s001.pdf]

SUPPLEMENTAL DATA

Preclinical Activity of Allogeneic SLAMF7-Specific CAR T-Cells (UCARTCS1) in Multiple Myeloma

Charlotte L.B.M. Korst[1,2], Chloe O'Neill[1,2], Wassilis S.C. Bruins[1,2], Meliha Cosovic[1,2], Inoka Twickler[1,2], Christie P.M. Verkleij[1,2], Diane Le Clerre[3], Maria Themeli[1,2], Isabelle Chion-Sotinel[3], Sonja Zweegman[1,2], Roman Galetto[3], Tuna Mutis[1,2], Niels W.C.J. van de Donk[1,2]

[1]Amsterdam UMC location Vrije Universiteit Amsterdam, Department of Hematology, De Boelelaan 1117, Amsterdam, Netherlands; [2]Cancer Center Amsterdam, Cancer Biology and Immunology, Amsterdam, The Netherlands; [3] Cellectis SA, 8 Rue de la Croix Jarry, 75013, Paris, France

Supplemental methods

UCARTCS1 Manufacturing

UCARTCS1 is manufactured from frozen mononuclear cells (MNCs) obtained from healthy volunteer donors. The T-cells are genetically modified to express a second generation CAR directed against SLAMF7, upon transduction with a lentiviral vector. Prior to transduction and CAR expression, the T-cell receptor alpha constant chain (TRAC) and the SLAMF7 genes are specifically disrupted by gene-editing using Transcription Activator Like Effector Nucleases (TALEN®). TALEN® are artificially engineered restriction enzymes capable of generating site-specific double-strand DNA breaks at a desired target site, leading to inactivation of the targeted gene. In the UCARTCS1 production process, the TRAC and SLAMF7 TALEN® are introduced into the cells as messenger Ribonucleic Acid (mRNA) using an electroporation system. This transient expression allows TALEN® to perform efficient targeted gene knock-out(s) before being degraded by the cells, preventing potential risks associated with long-term expression of nucleases in cells injected into patients. The cells are expanded in a bioreactor and, at the end of the UCARTCS1 manufacturing process, the remaining $TCR\alpha\beta^{+}$ cells are depleted ensuring minimal residual levels of $TCR\alpha\beta^{+}$ cells in the final product.

Although the TALEN® targeting the *TRAC* and *SLAMF7* genes were designed to minimize off-target cleavage by choosing target sites that differ from others in the human genome, a risk associated with TALEN®-mediated gene editing is off-target cleavage. The specificity of the TALEN® was assessed using an unbiased genome wide approach (Oligo Capture Assay, OCA) and Next Generation Sequencing (NGS). This analysis showed no cleavage activity above the background at the most putative OCA-predicted potential off-target sites in the TALEN®-treated T-cells. The presence of chromosomal aberrations that could be generated during production was evaluated by karyotype analysis. The only clonal aberration observed at low occurrence, and further confirmed by Fluorescence In Situ Hybridization (FISH) analysis, was a balanced translocation consistent with *TRAC/SLAMF7* rearrangements. These events are monitored on each GMP production run and FISH is performed if translocations are detected by karyotyping analysis to confirm *TRAC/SLAMF7* rearrangements. No other clonal abnormalities are accepted. Lastly, absence of *in vitro* aberrant proliferation, assessed by an IL-2 independent proliferation assay, is required before release of a batch for clinical investigation.

Supplemental Figure 1

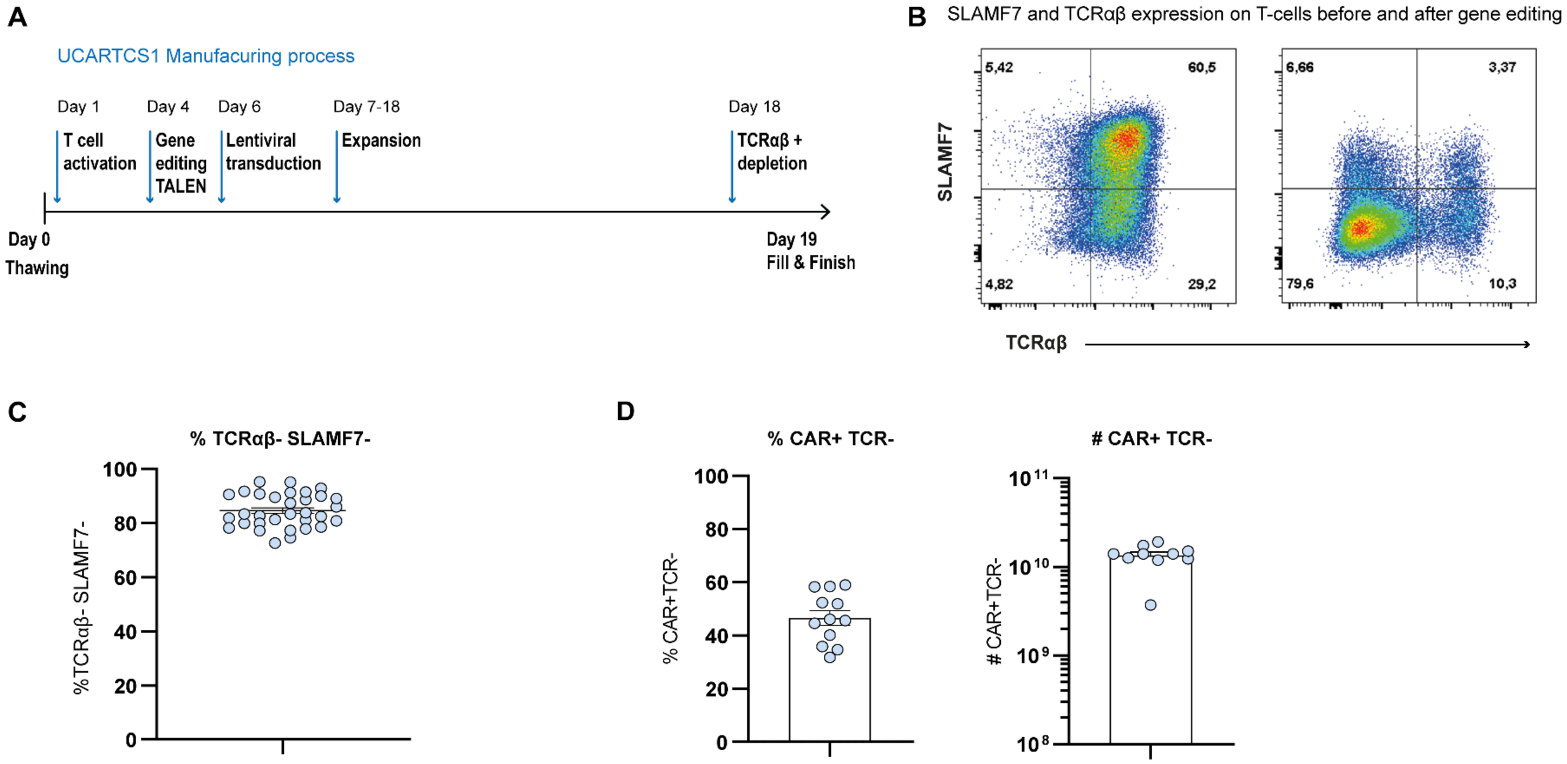

Supplemental Figure 1. UCARTCS1 production and characteristics of T cells

(A) Schematic overview of the 19-day UCARTCS1 production process. (B) SLAMF7 and TCRαβ expression by T-cells before electroporation of SLAMF7 and TRAC targeting TALEN mRNA (left panel) and 3 days after electroporation (right panel). (C) Percentage of SLAMF7/TCRαβ double negative T-cells assessed 3 days after electroporation of SLAMF7 and TRAC targeting TALEN mRNA (n=32). Data represent mean ± SEM. (D) Percentage of TCRαβ$^-$ CAR$^+$ cells at the end of the manufacturing process (left panel), and number of TCRαβ$^-$ CAR$^+$ cells at the end of the large scale manufacturing process (n=10, right panel). Data represent mean ± SEM.

Supplemental Figure 2

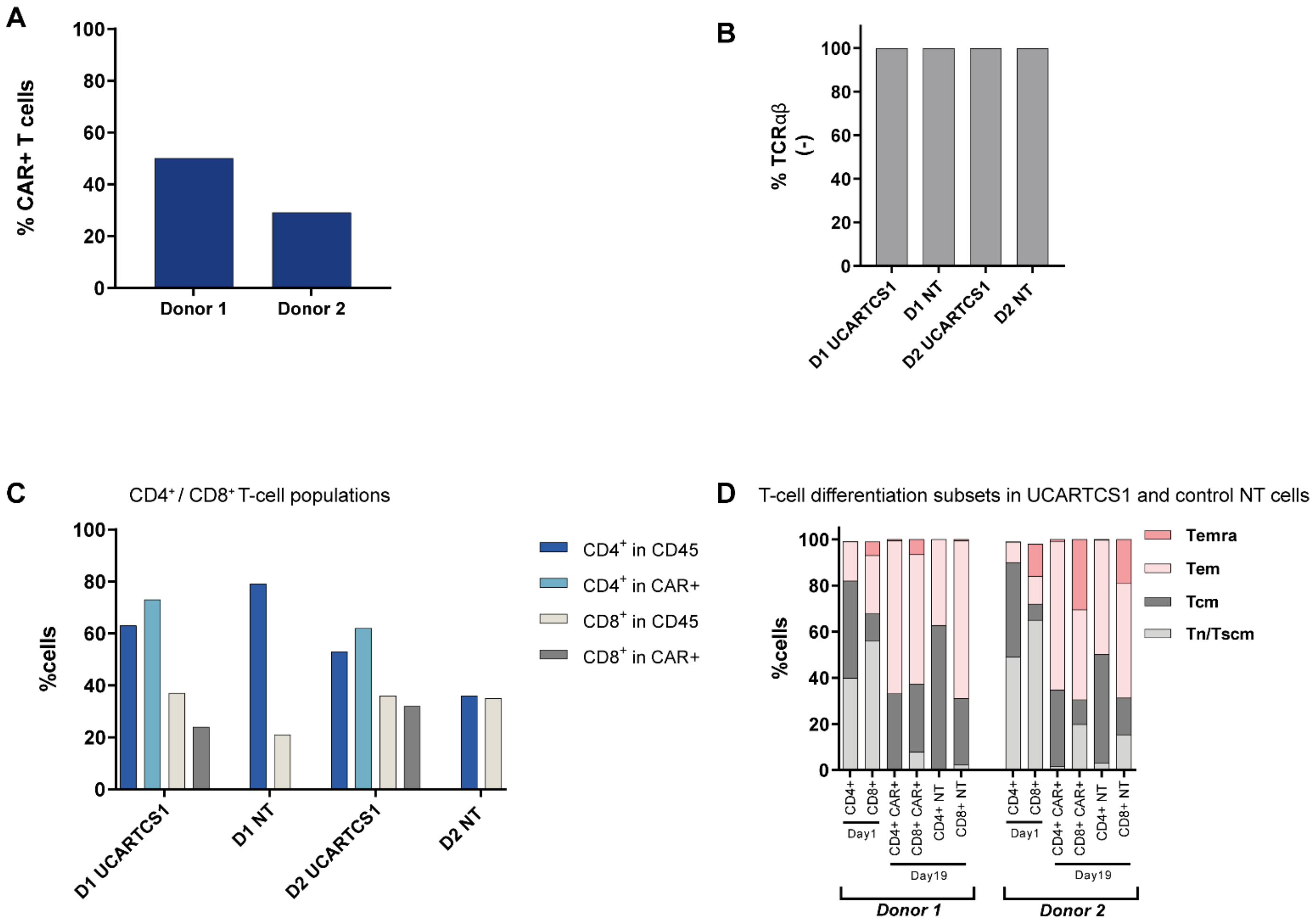

Supplemental Figure 2. Additional characterization of UCARTCS1 cells

UCARTCS1 cells and control (non-transduced (NT), SLAMF7/TCRαβ double knock-out) T-cells were generated from T-cells obtained from two different healthy donors. (A) The percentage of UCARTCS1 cells expressing the SLAMF7 CAR for donor 1 (D1) and donor 2 (D2). (B) UCARTCS1 cells and control NT T-cells were TCRαβ negative for both donors. (C) Percentage of $CD4^+$ (dark blue) or $CD8^+$ cells (light gray) in $CD45^+$ UCARTCS1 or control NT T-cells at the end of the production process (day 19). Light blue and dark gray bars depict percentage of $CD4^+$ or $CD8^+$ cells within the CAR^+ cells (for UCARTCS1 cells only). (D) T cell differentiation subsets at day 1 (at the beginning of UCARTCS1 production) in $CD4^+$ and $CD8^+$ cells from donor 1 and donor 2, and $CD4^+$ and $CD8^+$ T cell differentiation subsets at day 19 (end of manufacturing process) for donor 1 and donor 2 in the CAR^+ fraction of UCARTCS1 cells and in control NT T-cells. *Tcm, central memory T cells; Tem, effector memory T cells; TEMRA, terminally differentiated effector memory T cells expressing CD45RA; Tn/Tscm, naïve T cells and stem cell-like memory T cells.*

Supplemental Figure 3

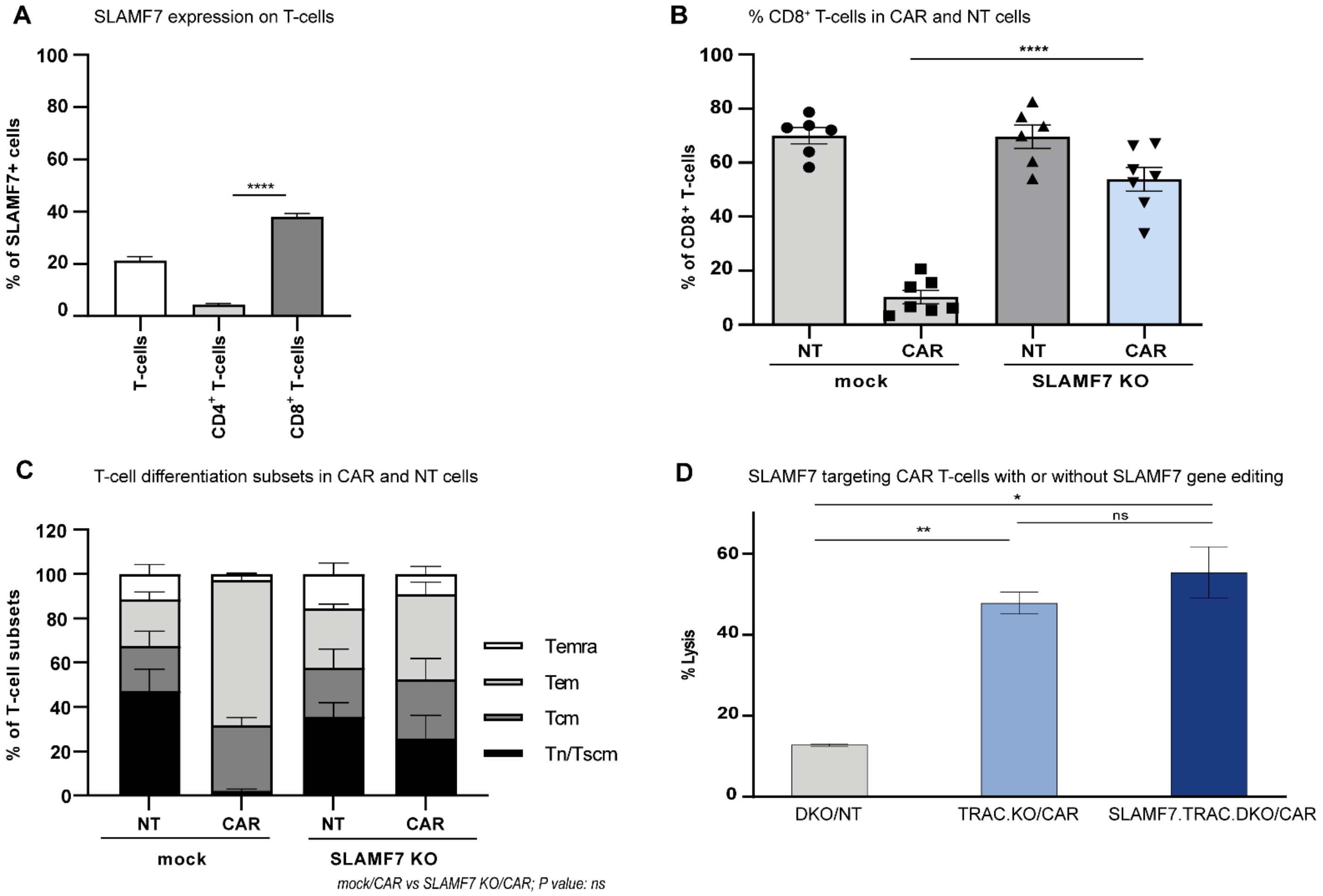

Supplemental Figure 3. Percentage of SLAMF7-positive T-cells and impact of SLAMF7 knockout on CD8+ T-cells

(A) SLAMF7 expression was assessed by flow cytometry on T-cells (CD4+ T-cells and CD8+ T-cells) 24 hours after thawing. Data represent mean ± SEM. Paired t-test was used to calculate significance between groups. (B) SLAMF7-targeting CAR T-cells were produced with or without SLAMF7 gene editing. At the end of the production process, flow cytometry was used to determine the percentage of CD8+ T-cells as a percentage of total T cells in which the SLAMF7 CAR was transduced in SLAMF7 knockout cells or in non-edited cells (mock). Non-transduced (NT) cells (SLAMF7-edited or not) were used as a control (n=6). Data represent mean ± SEM. Paired t-test was used to calculate significance between groups. (C) SLAMF7-targeting CAR T-cells were produced (small scale R&D grade productions) with or without SLAMF7 gene editing. At the end of the production process, flow cytometry was used to determine the percentage of T-cell differentiation subsets in SLAMF7 knockout cells or in non-edited cells

(mock) expressing the SLAMF7 CAR or not (NT cells) (n=3). Data represent mean ± SEM. Paired t-test was used to calculate significance between groups. D) Cytolytic capacity of SLAMF7-targeting CAR T-cells with or without SLAMF7 gene editing was assessed in a 4-hour flow cytometry-based *in vitro* cytotoxicity assay by using L363 cells loaded with a fluorescent dye as target cells (E:T ratio of 10:1). SLAMF7/TCRαβ double knock-out CAR T-cells were compared with TCRαβ knock-out CAR T-cells. Non-transduced , SLAMF7/TCRαβ double knock-out T-cells were used as control (DKO/NT). Data represent mean target cell lysis ± SEM of 3 independent experiments. Unpaired t-test was used to calculate significance between groups. *TRAC*, *TCRα constant chain gene; DKO, double knock-out; E:T ratio, effector to target-ratio; TCR, T-cell receptor; Tcm, central memory T cells; Tem, effector memory T cells; TEMRA, terminally differentiated effector memory T cells expressing CD45RA; Tn/Tscm, naïve T cells and stem cell-like memory T cells. ns, not significant; *, P < 0.05; **, P < 0.01; ***, P < 0.001; ****, P < 0.0001.*

Supplemental Figure 4

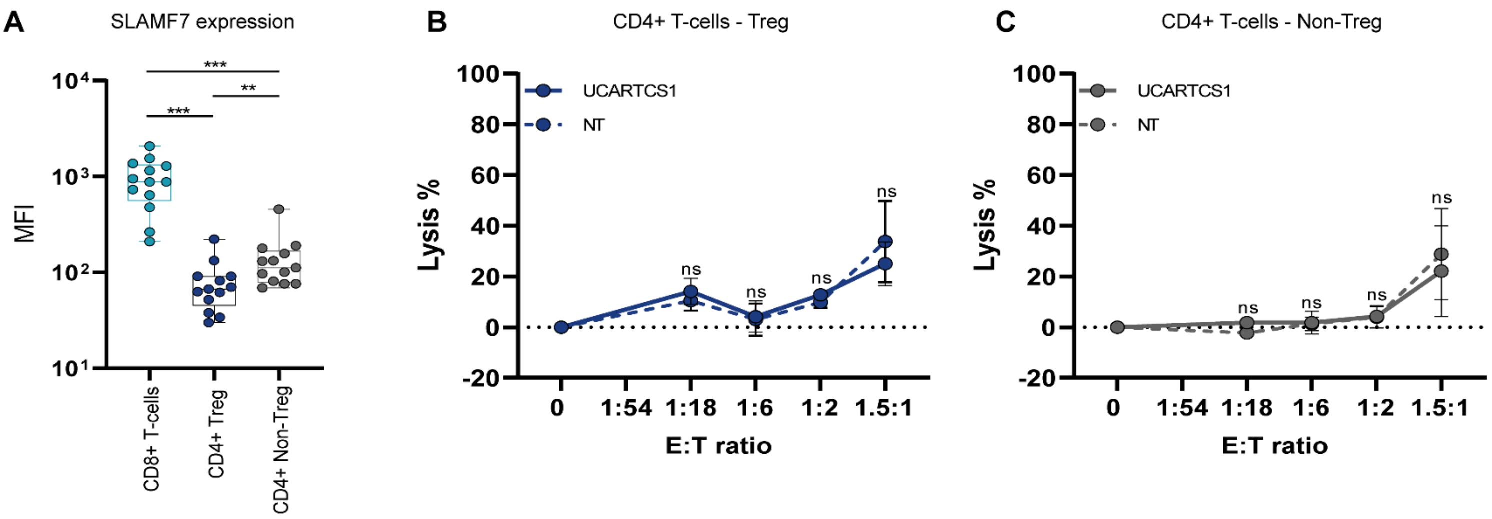

Supplemental Figure 4. Effect of UCARTCS1 on Tregs and $CD4^+$ non-Treg T-cells

A) SLAMF7 expression was evaluated on $CD8^+$ T-cells cells, $CD4^+$ Tregs, and $CD4^+$ T-cells not classified as Tregs in 13 BM samples from MM patients by flow cytometry. Tregs were defined as $CD3^+CD4^+CD25^+CD127^{-/low}$. Each dot represents an individual sample, with box and whiskers, representing median, 25th-75th percentile, and range. Groups were compared using Wilcoxon matched-pairs test. B-C) BM-MNCs obtained from 3 NDMM patients were incubated with UCARTCS1 cells or control (non-transduced (NT), SLAMF7/TCRαβ double knock-out) T-cells at different E:T ratios for 24 hours after which surviving $CD4^+$ T-cells (Tregs and $CD4^+$ T-cells not classified as Treg) were enumerated using flow cytometric analysis. Solid lines represent UCARTCS1 cells and dotted lines control NT T-cells. Data represent mean ± SEM. Groups were compared using Mann-Whitney U test. *BM-MNCs, bone marrow mononuclear cells; E:T ratio, effector to target-ratio; TCR, T-cell receptor; NDMM, newly diagnosed multiple myeloma; Treg, regulatory T cell; ns, not significant; *, P < 0.05; **, P < 0.01; ***, P < 0.001; ****, P < 0.0001.*

Supplemental Figure 5

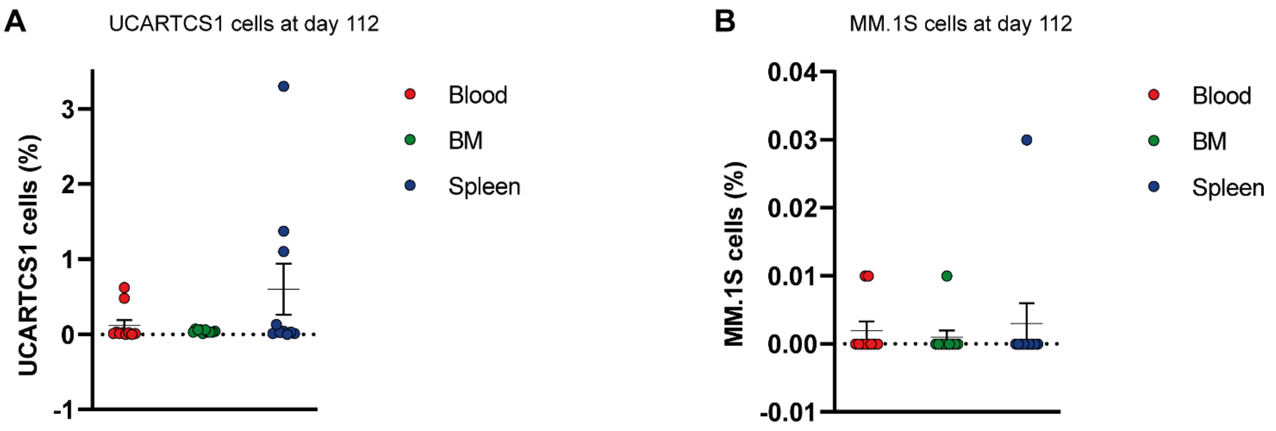

Supplemental Figure 5. Percentage of UCARTCS1 cells and MM.1S cells in mice at day 112

A-B) Shown are percentages of UCARTCS1 cells and MM.1S cells in the blood, BM and spleen of all 10 mice treated with $10x10^6$ UCARTCS1 cells at day 112 (end of study). UCARTCS1 cell percentage was assessed using flow cytometric analysis. UCARTCS1 cells were identified by the expression of human CD45 (hCD45) and by negativity for GFP. MM.1S-LUC-GFP tumor cells were identified as $GFP^+/hCD45^-$ cells. Data represent mean ± SEM. Each dot represents an individual mouse. *LUC, luciferase; ns, not significant; *, P < 0.05; **, P < 0.01; ***, P < 0.001; ****, P < 0.0001.*
